# Partial Internal Biliary Diversion in Progressive Familial Intrahepatic Cholestasis: Introduction of a New Approach

**DOI:** 10.5812/hepatmon.13549

**Published:** 2014-03-03

**Authors:** Seyed Abdollah Mousavi, Hasan Karami

**Affiliations:** 1 Department of Pediatric Surgery, Faculty of Medicine, Mazandaran University of Medical Sciences, Sari, IR Iran

**Keywords:** Cholestasis, Billiary Diversion, Pruritus

## Abstract

**Introduction:**

Facilitation of biliary salts secretion represents the mainstay of treatment for progressive familial intrahepatic cholestasis (PFIC). The purpose of this study was to introduce a new approach for the treatment of progressive familial intrahepatic cholestasis (PFIC) to avoid ostoma.

**Case Presentation:**

An 11-year-old girl with the diagnosis of PFIC underwent cholecystoappendicostomy with myotomy operation. Because of anastomosis stricture, she was reoperated with cholecystojejunocolic anastomosis and intussuscepted valve surgery. She was followed for 9 months. Despite disappointing outcomes of internal drainage with cholecystoappendicostomy, results of cholecystojejunocolic anastomosis with intussuscepted valve surgery were promising.

**Discussion:**

The cholecystojejunocolic anastomosis with intussuscepted valve surgery could be considered as a forthcoming approach in the treatment of intrahepatic cholestasis.

## 1. Introduction

Progressive familial intrahepatic cholestasis (PFIC) also known as Byler's disease, is an autosomal recessive disease leading to hepatic fibrosis, cirrhosis, and progressive hepatic failure (1) and is considered among the five leading causes of liver transplantation (2). Its estimated prevalence is 1:50000-100000 in general population (3). The disease occurs in early childhood and is diagnosed by clinical manifestations, ultrasound examination, liver biopsy, and specific tests to rule out other childhood cholestatic disorders (3). The pathologic features are nonspecific. Nonetheless, typical laboratory measures such as low to normal levels of gamma globulin transpeptidase (GGT), absence of lipoprotein X, low cholesterol, and high bile acids are usually observed (2). The pathophysiology seems to reside in hepatocellular damage resulting from concentrated biliary compounds (2). Severe pruritus is a disabling symptom in PFIC. Therefore, facilitating biliary salts extraction brings a major contribution to treatment (4). There is no treatment of choice for PFIC. Symptomatic treatment consists of ursodeoxycholic acid (UDCA), effective in only 60% of patients (5). Before 1990, the treatment of choice in unresponsive patients was liver transplantation (1). However, transplantation is largely limited by the availability of the organ and significant mortality and morbidity (6). Such obstacles have led to the introduction of diversion approaches to shortcut biliary compounds out of the liver. Nevertheless, the choice of surgical intervention is not agreed. Therefore, we decided to evaluate a new internal diversion approach in a patient diagnosed with PFIC.

## 2. Case Presentation

**Figure 1.**
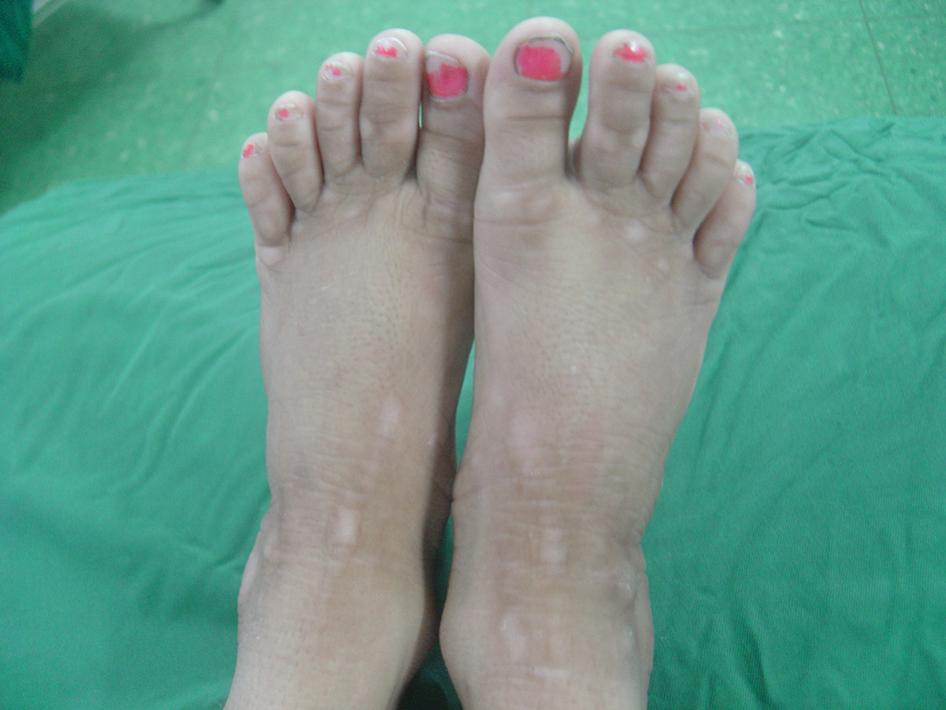
Feet Showing Broad Toe With Grossly Thickened and Lichenified Skin

An 11-year-old girl was born (weight of 3200g) as the second offspring of the related parents. She presented repeatedly, since 3 months of age, the symptoms of restlessness and icterus, which became intractable and unresponsive to common treatments. Laboratory work-up yielded no infectious etiology. Urine culture, sweat test, serum antinuclear antibody (ANA) levels, anti-dsDNA, anti-smooth muscle antibody, serum IgA, and anti-endomysial IgA levels were all in the normal range. Serum ceruloplasmin, gamma-glutamyl transferase and cholesterol had normal results. The bilirubin level was high and indicative of obstructive cholestasis. Abdominal ultrasonography revealed hepatomegaly with coarse surface and mild splenomegaly. The gall bladder contained multiple small stones. Analysis of repeated biopsies demonstrated mild to moderate ductal cholestasis and progressive biliary fibrosis in the last biopsy. On physical examination, the patient was short stature (height - 118 cm; weight - 22 kg; resulting a position under the third percentile for age), without mental retardation. Icterus was evidenced by excoriations all over the body, scaly skin with hyperpigmentation, and enlargement of fingers and toes (Figure 1). A diagnosis of Byler's disease was proposed, considering patient history and occurrence of icterus and severe pruritus at early age and also the biochemical and pathological evaluations. Therefore, the patient was recommended to undergo a biliary diversion surgery because of unresponsiveness to medical treatment.

### 2.1. Surgical Procedure

**Figure 2.**
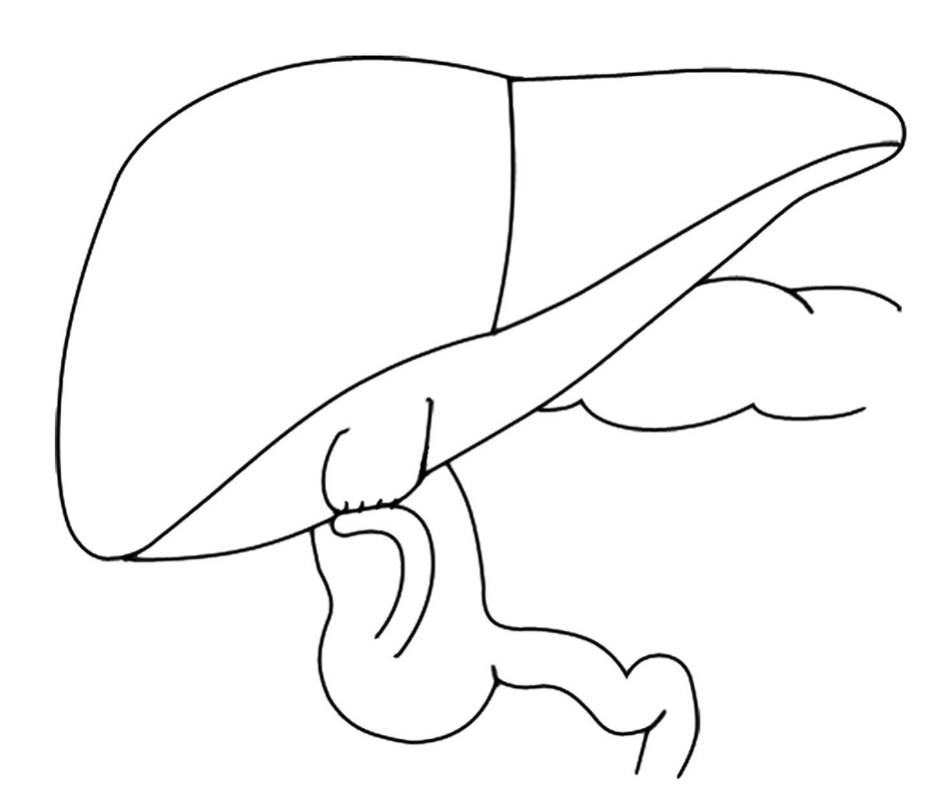
Cholecystoappendicostomy

We decided to perform an internal drainage procedure because of problems associated with ostoma placement in the external drainage. With respect to previous studies using the appendix for external drainage, we decided to evaluate its efficiency for the internal drainage (2, 7). The conditions were thoroughly explained to the patient's parents and after acquisition of informed consent, the operation was planned. After bowel preparation, an incision was made on the right upper quadrant. Grossly, the liver was hardened in texture, mildly enlarged with sharp edges and coarse surface. The gall bladder was enlarged with thickened wall and contained multiple stones. The cecum was free, and therefore was fixed beneath the liver to the abdominal wall. Then, the appendix tip was cut and cleaned with a 12F catheter. A longitudinal myotomy was performed from the appendix tip towards the cecum. Then, a longitudinal 3 cm long incision was made in the tip of appendix, similarly to the gall bladder fund us, to avoid stricture at the site of anastomosis. All the visible stones were extracted and a single layer cholecystoappendicostomy was performed using a Vicryl 5/0 suture (Figure 2). 

**Figure 3.**
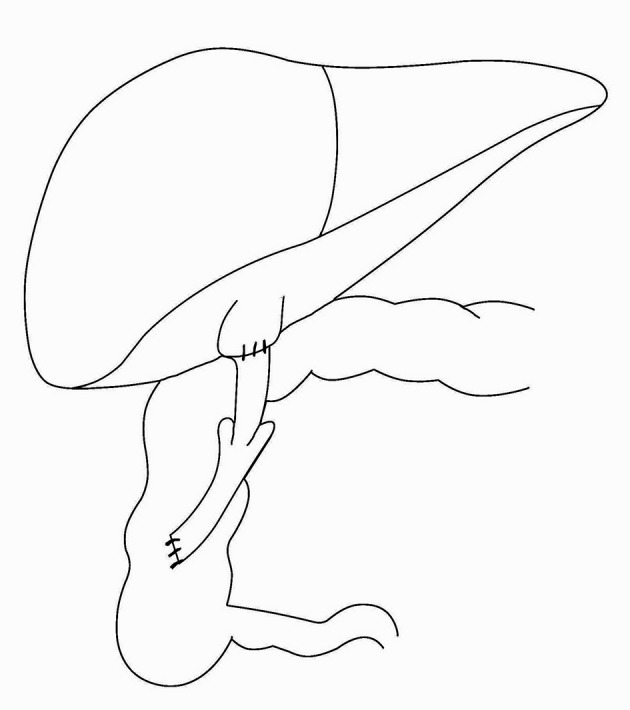
Cholecystojejunocolic Anastomosis

One day after the procedure, the patient could tolerate oral feeding and dismissed. After 48 hours, her icterus and pruritus were alleviated. Stool was green and loose. However, 15 days after the operation, pruritus and icterus returned gradually and stool appearance was the same as before the operation indicating treatment failure. The patient was recommended to be reoperated with a different approach in a month's time. The abdomen was incised transversally at the previous cut. A significant stricture at the anastomosis site was the reason for failure and led to appendectomy. To resume internal diversion, a 15 cm long jejunal segment was excised and installed between the proximal gall bladder and the lower segment of the anterior ascending colon with is operistaltic approach, using a single layer of Vicryl 4/0. Then, a3 cm long invagination was made at mid jejunum to prevent colon regurgitation into gall bladder (Figure 3). The abdominal wall was closed conventionally and antibiotic prophylaxis administered (cefazolin 50 mg/kg/day). Two days after the operation, the child was completely asymptomatic. After 4 days, she began oral feeding and, on day 5, she was discharged. She passed green stool once daily. Low-dose cefixime was administered for a month. During a 9-month follow-up, she was well, except for a transient self-remitting abdominal colicky pain, and the pruritus was completely relieved. Stool was green and loose and she gained about 4 kg.

## 3. Discussion

The PFIC constitutes 10-15% of pediatric cholestasis and is a reason for pediatric liver transplantation in 10-15% of candidates (3). In addition to hepatocellular damage, it causes significant pruritus and failure to thrive. Besides conventional medical management, biliary diversion does not only improve symptoms, but also reduces the associated risks of hepatic failure for those waiting for organ transplantation. Diversion techniques are classified into three categories:

1) Partial extrabiliary diversion (PEBD) using appendix or jejunum (2, 4, 6, 7).

This technique is useful as a bridge for alleviating icterus and pruritus for those waiting for trans-plant. However, it is associated with ostoma complications, such as prolapse or reflux, infection, and high-volume bile excretion (2, 6). Rebhandl et al. (2) reported that the bile volume excretion is 600-1200 mL daily, which is very disturbing. About one-third of patients experienced moderate to severe dehydration and hyponatremia during the first 2 weeks after the operation, as a result of bile excretion (5).

2) Ilealbypass surgery.

This is criticized because of choleretic-type diarrhea and uncertain long-term results. Furthermore, there are reports of late relapse, possibly because of gradual ileum accommodation (8, 9).

3) Partial internal biliary diversion (PIBD) using cholecystojejunocolic approach (1, 10).

Using the latter technique, bile acids in the intrahepatic cycle could be reduced to up to 50% (i.e. 50% reduction in the canaliculi preload) (11). This technique requires three anastomoses and is complicated by intermittent diarrhea due to high concentration of bile salts in the colon (10). However, it avoids ostoma complications and is well-tolerated by patients. We chose to divert the bile current into the colon via the appendix and only with one anastomosis, as the appendix has been continuously used as a substitute for biliary ducts and tolerates bile well (2).As we were concerned for the postoperative stricture, we dilated the anastomosis site and performed myotomy to ablate the muscular tone. Despite all these implementations, the stricture occurred right at the junction of the gall bladder. In the reoperation procedure, we performed the method of Bustorff-Silva et.al (1) through the jejunum to internally divert the bile. However, we selected a longer jejunalsegment and made a longitudinally invagination to prevent colon regurgitation. Although the symptoms were resolved completely by this approach, the three anastomoses requirement is a major disadvantage. The partial internal biliary diversion with cholecystojejunocolic anastomosis and intussuscepted valve could be considered as an appropriate approach for the treatment of intrahepatic cholestasis. However, we believe that working more on cholecystoappendicostomy and resolving its deficiencies may lead to a more frequent utilization of this technique in future. We suggest the reduction of appendix length, dilation of the anastomosis site, and/or insertion of a catheter in its lumen.

## References

[1] Bustorff-Silva J, Sbraggia Neto L, Olimpio H, de Alcantara RV, Matsushima E, De Tommaso AM (2007). Partial internal biliary diversion through a cholecystojejunocolonic anastomosis--a novel surgical approach for patients with progressive familial intrahepatic cholestasis: a preliminary report.. J Pediatr Surg..

[2] Rebhandl W, Felberbauer FX, Turnbull J, Paya K, Barcik U, Huber WD (1999). Biliary diversion by use of the appendix (cholecystoappendicostomy) in progressive familial intrahepatic cholestasis.. J Pediatr Gastroenterol Nutr..

[3] Yang H, Porte RJ, Verkade HJ, De Langen ZJ, Hulscher JB (2009). Partial external biliary diversion in children with progressive familial intrahepatic cholestasis and Alagille disease.. J Pediatr Gastroenterol Nutr..

[4] Whitington PF, Whitington GL (1988). Partial external diversion of bile for the treatment of intractable pruritus associated with intrahepatic cholestasis.. Gastroenterology..

[5] Jacquemin E, Hermans D, Myara A, Habes D, Debray D, Hadchouel M (1997). Ursodeoxycholic acid therapy in pediatric patients with progressive familial intrahepatic cholestasis.. Hepatology..

[6] Halaweish I, Chwals WJ (2010). Long-term outcome after partial external biliary diversion for progressive familial intrahepatic cholestasis.. J Pediatr Surg..

[7] Gauderer MW, Boyle JT (1997). Cholecystoappendicostomy in a child with Alagille syndrome.. J Pediatr Surg..

[8] Kalicinski PJ, Ismail H, Jankowska I, Kaminski A, Pawlowska J, Drewniak T (2003). Surgical treatment of progressive familial intrahepatic cholestasis: comparison of partial external biliary diversion and ileal bypass.. Eur J Pediatr Surg..

[9] Hollands CM, Rivera-Pedrogo FJ, Gonzalez-Vallina R, Loret-de-Mola O, Nahmad M, Burnweit CA (1998). Ileal exclusion for Byler's disease: an alternative surgical approach with promising early results for pruritus.. J Pediatr Surg..

[10] Ganesh R, Suresh N, Sathiyasekeran M, Ramachandran P (2011). Partial internal biliary diversion: a solution for intractable pruritus in progressive familial intrahepatic cholestasis type 1.. Saudi J Gastroenterol..

[11] Melter M, Rodeck B, Kardorff R, Hoyer PF, Petersen C, Ballauff A (2000). Progressive familial intrahepatic cholestasis: partial biliary diversion normalizes serum lipids and improves growth in noncirrhotic patients.. Am J Gastroenterol..

